# Contributions of direct versus indirect mechanisms for regulatory dendritic cell suppression of asthmatic allergen-specific IgG1 antibody responses

**DOI:** 10.1371/journal.pone.0190414

**Published:** 2018-01-02

**Authors:** Yanna Ma, Wojciech Dawicki, Xiaobei Zhang, John R. Gordon

**Affiliations:** Division of Respirology, Critical Care and Sleep Medicine, Department of Medicine, University of Saskatchewan, Saskatoon, Saskatchewan, Canada; Université Paris Descartes, FRANCE

## Abstract

IL-10-differentiated dendritic cells (DC10) can reverse the asthma phenotype in mice, but how they suppress the asthmatic B cell response is unclear. Herein we assessed the mechanism(s) by which DC10 and DC10-induced Treg affect IgG1 production in asthma. We observed a rapid decline in lung-resident OVA-specific IgG1-secreting B cells on cessation of airway allergen challenge, and intraperitoneal DC10 therapy did not amplify that (p>0.05). It did however increase the loss of IgG1-B cells from the bone marrow (by 45+/-7.2%; p≤0.01) and spleen (by 65+/-17.8%; p≤0.05) over 2 wk. Delivery of OVA-loaded DC10 directly into the airways of asthmatic mice decreased the lung IgG1 B cell response assessed 2 dy later by 33+/-9.7% (p≤0.01), while their co-culture with asthmatic lung cell suspensions reduced the numbers of IgG1-secreting cells by 56.5+/-9.7% (p≤0.01). This effect was dependent on the DC10 carrying intact allergen on their cell surface; DC10 that had phagocytosed and fully processed their allergen were unable to suppress B cell responses, although they did suppress asthmatic Th2 cell responses. We had shown that therapeutic delivery of DC10-induced Treg can effectively suppress asthmatic T and B cell (IgE and IgG1) responses; herein CD4^+^ cells or Treg from the lungs of DC10-treated OVA-asthmatic mice suppressed *in vitro* B cell IgG1 production by 52.2+/-8.7% (p≤0.001) or 44.6+/-12.2% (p≤0.05), respectively, but delivery of DC10-induced Treg directly into the airways of asthmatic mice had no discernible impact over 2 dy on the numbers of lung IgG1-secreting cells (p≥0.05). In summary, DC10 treatment down-regulates OVA-specific B cell responses of asthmatic mice. While DC10 that carry intact allergen on their cell surface can dampen this response, DC10-induced Treg are critical for full realization of this outcome. This suggests that infectious tolerance is an essential element in regulatory DC control of the B cell response in allergic asthma.

## Introduction

Allergic asthma is a chronic immunoinflammatory condition of the airways, wherein allergen-specific type 2 helper T (Th2) cells drive B cell isotype switching to IgE and IgG1 antibodies, and also the eosinophilic inflammatory response that is pathognomic of this disease. Allergen-specific IgE and IgG1 antibodies are substantially elevated in asthmatic individuals and that is seen also in mouse models of asthma [1,2,3]. IgE and IgG1 antibodies reportedly play distinct roles in the pathogenesis of allergic diseases, including asthma and anaphylaxis related to food allergies [4,5]. IgE sensitizes mast cells and basophils for degranulation following allergen cross-linking of IgE-occupied Fc-epsilon-RI [6], while IgG1 antibodies are thought to form immune complexes with allergen within the lungs, thereby recruiting downstream asthma-associated innate cells such as mast cells, basophils, and eosinophils that carry activating Fc-gamma receptors (i.e., in mice, Fc-gamma-R1, -RIII and–RIV) [4,5].

Conventional treatments for asthma are largely symptom-based, targeting respiratory inflammation and bronchoconstriction responses, rather than the immunologic basis of this disease. Recent advances have shown that immune tolerance can be established in mouse models of asthma by use of regulatory dendritic cells (DCreg) [7,8,9]. Thus, differentiation in the presence of IL-10, for example, induces a tolerogenic or regulatory phenotype in both human monocyte- and murine bone marrow-derived DC (DC10) [10,11,12,13]. Such DC10 express elevated levels of IL-10 and TGF-ß, and low levels of MHC II and costimulatory signals [9,11,14]. DC10 treatment reverses airway hyperresponsiveness and airway Th2 recall responses to allergen challenge, and reduces the levels of circulating allergen-specific IgG1 and IgE in ovalbumin (OVA) [8,14,15,16] and house dust mite (HDM) [9] mouse models of asthma. It also induces Th2 cells in treated mice to transdifferentiate into CD4^+^CD25^+^Foxp3^+^ regulatory T cells (Treg) [9,11,14]. DC10 generated from monocytes of atopic asthmatic donors can similarly induce allergen tolerance among autologous Th2 cells from these donors [11]. In a manner analogous to DC10, retinoic acid-differentiated DC can reverse food allergen (e.g., peanut) sensitivity in mouse models, ameliorating anaphylactic responses to allergen challenge and also reducing allergen-specific IgE and IgG1 levels in fully hypersensitive mice, albeit via distinct mechanisms [17].

Precisely how DC10 treatments reduce allergen-specific IgG1 responses in asthmatic mice has not been explored. We know that DC10 suppress Th2 cell responses [8,9], but as noted they also induce differentiation of CD25^+^Foxp3^+^ Treg [14]. Thus, in principle DC10 could affect B cell response through simple attenuation of Th2 cell help, but also through more focused means such as via direct interactions between the B cells and either DC10 themselves and/or the Treg that DC10 treatments induce [9,11,14]. There is certainly precedent for direct and productive interactions between antigen-laden DC and B cells. For example, DC that have acquired antigens through phagocytosis of either anti-DC immunoreceptor-2/antigen complexes [18] or environmental antigens [19] can subsequently present intact antigen directly to B cells, thereby inducing robust B cell responses [19]. In contrast, immature mouse DC suppress BCR signaling-induced proliferation of B cells in a contact-dependent fashion [20], while CD25^+^Foxp3^+^ natural Treg can reportedly directly suppress B cell responses [21,22].

In this study, we explored the extent to which DC10 suppress the allergen-specific IgG1 response across multiple anatomic compartments in a mouse model of OVA-asthma, but also the mechanisms by which they do so. Our data indicates that OVA-specific IgG1 secretion by B cells can be down-regulated directly by DC10 that present intact allergen on their plasma membrane, but not by DC10 that present fully processed allergen. We also show that DC10-induced Treg can directly suppress the B cell response, and that agrees with previous data showing that DC10-induced Treg are potent regulators of asthmatic T and B cell responses.

## Materials and methods

### Animals, reagents, and materials

Female BALB/c mice were purchased from Charles River Laboratories (Sherbrooke, PQ), while OVA-T cell receptor transgenic OT II mice were from the Jackson Laboratories (Bar Harbor, ME). All mouse studies were approved by the University of Saskatchewan Animal Research Ethics Board, in accord with the guidelines of the Canadian Council on Animal Care. The materials and reagents used and their sources have largely been reported [9,14,16], but included: MHC II-restricted OVA peptide_323-339_ (InvivoGen, San Diego, CA); [methyl-^3^H] thymidine (American Radiolabeled Chemicals Inc, St. Louis, MO); CD16/CD32 (‘Fc block’) antibodies (BD Pharmingen Inc, Mississauga, ON); PE-conjugated anti-mouse CD25, FITC-conjugated anti-mouse CD4, PEcy5-conjugated anti-mouse CD19, APC-conjugated anti-mouse Foxp3, and their appropriate isotype controls (eBioscience Inc., San Diego, CA); Prolong Diamond Anti-fade Mountant with DAPI (for nuclear staining on confocal slides; Thermo Fisher Scientific, Molecular Probes); mouse CD4^+^ T cell magnetic sorting kits, mouse CD19 microbeads and CD4^+^CD25^+^ regulatory T cell isolation kits (Miltenyi Biotec, Auburn, CA); intracellular Foxp3 staining kits (eBioscience Inc, San Diego, CA); Elispot MultiScreen_HTS_ IP 96-well plates (Millipore Ltd, Etobicoke, ON); streptavidin-alkaline phosphatase conjugate (Invitrogen Life Technologies Inc, Burlington, ON); and nitro-blue tetrazolium [NBT]/5-bromo, 4-chloro, 3-indolylphosphate [BCIP] stock solution (Roche Diagnostics, Mannheim, Germany).

### Generation of dendritic cells

Dendritic cells were generated from bone marrow cells as reported previously [9,23]. Briefly, bone marrow cells obtained from the tibiae and femurs of mice were differentiated by culture in recombinant murine (rm) GM-CSF in RPMI 1640, 1% antibiotics/antimycotics, 50 μM 2-mercaptoethanol, and 10% FBS (complete medium) for 10 or 7 days, and then further cultured for 1 or 3 dy with 1 μg/ml lipopolysaccharide (LPS)(*Escherichia coli* serotype 0127: B8; immunostimulatory DC-LPS) or rmIL-10 (50 ng/ml; DC10) respectively. The cells were then pulsed with 50 μg/ml OVA overnight at 37°C and washed 3 times prior to being used [14]. In some experiments the cells were pulsed instead with MHC II-restricted OVA peptide_323-339_ for 2 h, then washed as above before being used.

### Asthma models and DC treatments

**Fig 1:**
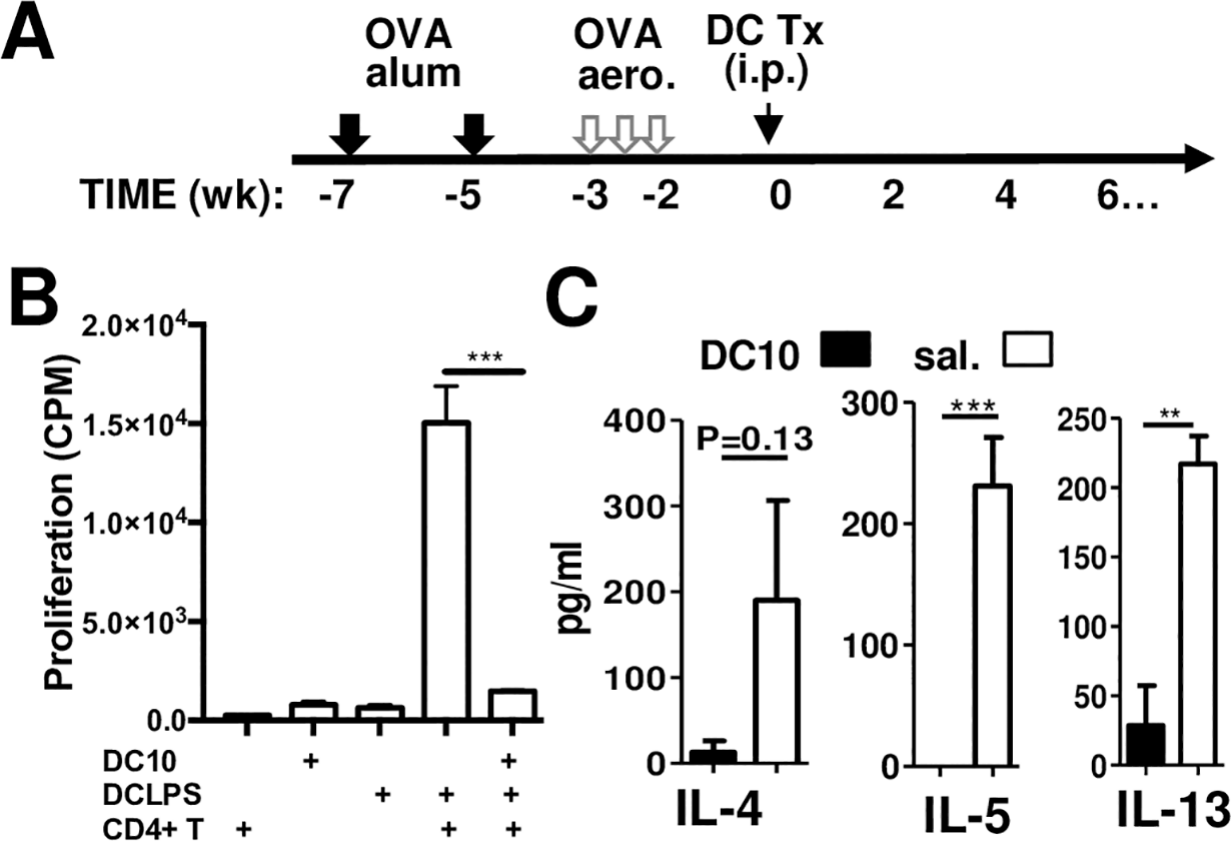
DC10 can suppress asthmatic Th2 responses *in vitro* and *in vivo*. IL-10-differentiated (DC10) or immunostimulatory (DC-LPS) DC were generated by culture of BALB/c mouse bone marrow cells with rmGM-CSF, and differentiated further by addition of rmIL-10 or LPS (*Escherichia coli* serotype 0127: B8), respectively. Cells were pulsed with OVA for another 24 h and washed prior to use. (**A)** Asthma was induced in BALB/c mice by i.p. administration of OVA-alum and repeated exposure to OVA aerosols, as noted in the Materials and Methods. The asthmatic mice were treated with saline or DC10 on dy 0. (**B)** CD4^+^ T cells were magnetically sorted from the spleens of OVA-TCR transgenic OTII mice. DC10 and DC-LPS were generated as noted in the Materials and Methods section and co-cultured with the OTII CD4^+^ T cells in the presence of OVA_323-339_ peptide, and on day 3 cell proliferation was assessed using ^3^H-thymidine uptake assays. Control wells contained DC10, DC-LPS, or OTII CD4^+^ T cells alone. This data is representative of three identical experiments, with four replicate wells in each group; each bar represents the mean (±SEM) of the 4 wells. (**C)** Three weeks after saline- or DC10-treatment, asthmatic BALB/c mice were re-challenged for 20 min with nebulized OVA aerosols and then sacrificed 2 dy later to assess Th2 cytokine levels in their broncholaveolar lavage fluids by ELISA. Each bar represents the mean (±SEM, n = 3 or 5 mice/group), with duplicate wells/sample. The data presented are representative of three experiments. The statistical analyses were performed using one-way ANOVA assays with Tukey’s *post-hoc* testing. ** and *** signify p<0.01 and <0.001, respectively.

The establishment of the asthma phenotype in mice has been described previously [3,24], and is presented schematically in Fig 1A. Briefly, BALB/c mice were given 2 μg OVA on 1 mg of alum (i.p.) on wk -7 and -5, and then from wk -3 to wk -2 they were exposed every second day via the airway to nebulized aerosols of 1% OVA (20 min/dy). Two weeks after the last airway challenge, the asthmatic mice were treated (i.p.) with 1x10^6^ OVA-presenting DC10 and then sacrificed for assessment at varying times thereafter; all mice were given a 20 min recall allergen challenge with nebulized 1% OVA 2 dy before sacrifice and were sacrificed by exanguination under surgical level anaesthesia with isofluorane. After sacrifice, bronchoalveolar lavage (BAL) was done on the mice, as noted [16], and the BAL fluids analyzed for Th2 cytokines. In one set of experiments we recovered cells from the peritoneal cavities of our mice by peritoneal lavage with 3 ml volumes of complete medium, and assessed the numbers of OVA-specific IgG1- and IgA-secreting cells by ELISPOT assay.

### FACS characterization of T and B cells

For cell surface staining, T cells and B cells were pre-incubated at room temperature with 0.5 μg Fc block for 10 min, and then incubated for 20 min on ice with the marker-specific (e.g., anti-mouse B220, CD4, CD19, CD25, CD138) or appropriate isotype control antibodies. To analyze intracellular Foxp3 expression, cells were permeabilized overnight at 4°C with the antibody suppliers’ fixation and permeabilization buffer, washed thoroughly and treated with Fc block for 10 min, as above, and then incubated with the anti-Foxp3 antibodies for 20 min on ice, before washing and subsequent analysis by FACS, using a FACScan (Becton Dickinson, Mountain View, CA); 5x10^4^ events were acquired in each sample. The flow cytometry data was analyzed by FlowJo 8.5.2 analysis software (Tree Star Inc, Ashland, OR). Cell gating was based on isotype control antibodies compared to positive staining, as noted in individual figure captions.

### Confocal microscopy and FACS assessment of cell surface allergen

DC10 were pulsed with OVA-FITC (50 μg/ml) either overnight or for 2 h at 4 or 37°C and washed twice with complete medium; some of the washed cells were further cultured overnight at 37°C to allow full allergen processing and then washed again. For confocal or FACS analysis the cells were pre-incubated with 0.5 μg Fc block for 10 m, then stained for 20 min on ice with PE anti-mouse CD11c (or isotype control) antibodies and washed three times with PBS before fixation with 4% paraformaldehyde (20 min, 20°C). Samples of the cells (5x10^4^/slide) were applied to cytocentrifuge slides and counter-stained with the nuclear stain DAPI, and kept in the dark at room temperature for 24 h prior to confocal analysis. Confocal pictures were captured using a Zeiss LSM 700 microscope. The fixed cells were also analyzed by FACS using a FACScan flow cytometer (Beckman-Dickinson) and analyzed by FlowJo 8.5.2 software.

### CD4^+^ T cell isolation or depletion, and CD4^+^CD25^+^ regulatory T cell and CD19^-^ lung cell isolation

To generate single cell suspensions, the lungs were removed from each mouse, minced mechanically, and digested for 90 min at 37°C in 0.75 mg/ml collagenase and 2 mg/ml hyaluronidase in HBSS/10% FCS. The mononuclear cells were purified from the dispersed populations by density gradient centrifugation and washed in complete medium before use, as noted [3]. CD4^+^ T cells were positively-selected from splenocytes of otherwise untreated asthmatic OT II mice using CD4-specific paramagnetic beads; these beads were also used for depletion of CD4^+^ cells (i.e., T helper cells) from asthmatic lung single cell suspensions. For regulatory T cell isolation, lung single cell suspensions from DC10-treated asthmatic mice were depleted of non-CD4^+^ cells by negative selection magnetic sorting, and then the CD25^hi^ cells were positively selected from the residual CD4^+^ T cells. To generate lung CD19^-/lo^ plasma cell-enriched populations, CD19^hi^ cells were removed from asthmatic lung single cell suspensions using specific paramagnetic beads, all according to the supplier’s protocols.

### Generation of DC10-induced Treg *in vitro* and purification of natural Tregs

CD4^+^ effector T (Teff) cells, which we have previously reported to secrete IL-4, -5, -9 and -13, and to be CD44^hi^CD69^+^CD62L^lo^ [25] were purified from the lungs of asthmatic mice by positive-selection magnetic sorting and co-cultured with DC10 (3x10^5^ DC10 and 1x10^6^ Teff/ml) in 6-well plates in the presence of rm-IL-2 (10U/ml). After 5 dy the CD4^+^CD25^+^ Treg were positively-selected from the co-cultures using a magnetic Treg sorting kit. Natural Treg were positively selected from the lungs and spleens of naive BLAB/c mice as a control for DC10-induced Tregs.

### Proliferation and suppression assays

Optimization of our DC10 suppression assay has been reported previously (14). In brief, half-maximal numbers of OVA_323-339_ peptide (3 μM)-pulsed immunostimulatory DC-LPS (3.7x10^3^ cells/well) and 1x10^5^ CD4^+^ T cells/well were co-cultured for three days as our baseline Teff cell proliferative response. The indicated numbers of DC10 were added to these DC-LPS/Th2 cell co-cultures to assess their impact on Th2 cell proliferative responses; splenocytes from normal mice were added in sufficient numbers to ensure that all wells contained equal numbers of cells. ^3^H-thymidine (0.5 μCi/well) was added for the final 24 h of culture, and then the cellular DNA was harvested onto glass-fibre filters and levels of ^3^H-thymidine incorporation determined by liquid scintillation counting (Beckman Coulter LS6500 Liquid Scintillation Counter).

### ELISA

Heparin-anti-coagulated plasma was obtained by cardiac puncture. A reference standard of archived pooled plasma from multiply OVA/alum-sensitized asthmatic mice (n = 5) was used for the OVA-specific IgE, IgA and IgG1 assays, while plasma from saline-treated asthmatic or normal mice were used as positive or negative controls, respectively. The experimental samples were diluted 1:100 in PBST. For the OVA-specific IgA and IgG1 ELISA, 96-well flat-bottom immunolon-4 plates were coated with OVA (10 μg/ml coating buffer [1M NaHCO_3_, 1M Na_2_CO_3_; pH 9.6]) as a capture reagent and bound OVA-specific antibodies were detected using biotinylated anti-mouse IgA or IgG1 (1 μg/ml). For the OVA-specific IgE assays, the wells were coated with anti-IgE (1 μg/ml) to capture sample IgE antibodies, and biotinylated OVA was used to detect the captured IgE. The plates were read at a wavelength of 405 nm on an ELISA plate reader. BAL fluids were analyzed for the indicated cytokines as noted previously [16].

### ELISPOT assay

Polyvinylpyrrolidone (PVDF)-free membrane ELISPOT plates were coated overnight at 4°C with 0.5% OVA in coating buffer. The wells were blocked for 2 h at 37°C with 200 μl/well of complete medium, washed, and 1x10^5^, 2x10^5^ or 5x10^5^ cells in 200 μl volumes of complete medium were added to each well and incubated for 5 h at 37°C. In all assays, after the 5 h incubation period the cells were lysed by the addition for 1 min of 100 μl/well distilled H_2_O. Biotinylated anti-mouse IgA or IgG1 (1 ng/ml in PBS), used to detect IgA or IgG1 antibody-secreting B cells, respectively, was added to the plates overnight at 4°C. Streptavidin-alkaline phosphatase enzyme conjugate (diluted 1:5000) was added for 1.5 h at room temperature, and BCIP/NBT plus substrate (1:50 dilution in 0.1M Tris-HCl pH 9.5, 0.1M NaCl, 0.05M MgCl_2_) was used to develop the spots, which were counted in a blinded fashion using an Olympus binocular microscope (SZX9; Tokyo, Japan). All wells were washed extensively with PBST to remove residual reagent between each step. The data are expressed as the numbers of OVA-specific IgA- or IgG1-secreting B cells per 1x10^6^ input cells. We confirmed in preliminary experiments that there was a linear relationship between the numbers of input cells and the numbers of antibody-secreting cells in our ELISPOT assays (S1 Fig; r^2^ = 0.89).

### Impact of local DC10 or DC10-iTreg on pulmonary IgG1 responses in asthmatic mice

Asthmatic BALB/c mice were given 1x10^6^ OVA-pulsed DC10 (OVA-DC10) or magnetically-sorted CD25^+^ DC10-induced Treg, delivered into the airway by transtracheal instillation. Control mice were given equal numbers of house dust mite allergen-pulsed DC10 or CD25^+^ natural Treg from untreated normal mice. Two days after the treatment, the lung and lung-draining (mediastinal) lymph node cells were collected from the recipients to assess B cell OVA-specific IgA or IgG1-secretion, as determined by ELISPOT.

### Statistics

All data were analyzed using GraphPad Prism 5.0 (GraphPad Software Inc., La Jolla, CA). The normality of the data was determined and then statistical significance was assessed using t tests, one-way ANOVA assays with Tukey’s *post-hoc* testing or linear regression analysis. *, **, *** and **** signify P<0.05, <0.01, <0.001, and <0.0001 respectively, versus the indicated controls.

## Results

### DC10 can promote Th2 cell and asthma tolerance

We had previously documented that DC10 express low levels of cell surface MHCII, CD40, CD80, CD86, and secrete low levels of IL-12 but rather more IL-10, relative to immunostimulatory DC [9,26]. We also previously reported that while DC10 are effective in dampening the asthma phenotype in mice, including significantly reducing the plasma levels of OVA-specific IgG1 [16], neither immature or TNF-matured OVA-presenting DC or irrelevant allergen-presenting DC10 are effective in reversing the asthma phenotype in mouse models (16). Similarly, irrelevant allergen-presenting human DC10 do not significantly ameliorate asthmatic human Th2 responses *in vitro* [3]. Herein we confirmed the regulatory functions of our DC10, first by examining their abilities to suppress DC-LPS-induced asthmatic Th2 cell proliferation *in vitro*. The overall protocol for the induction of OVA-asthma [3] and the DC10 treatment [16] is depicted in Fig 1A. We co-cultured OVA-pulsed DC10 with OVA-presenting stimulatory DC-LPS and magnetically-sorted Th2 cells (≥90% CD4^+^; data not shown) from asthmatic mice, and found that the DC10 did suppress DC-LPS-induced Th2 cell proliferation (Fig 1B). In order to confirm that our OVA-DC10 were also tolerogenic *in vivo*, we used them to treat mice in which we had induced an asthma phenotype, and here too OVA-presenting DC10 significantly down-regulated 2 dy airway Th2 (i.e., IL-4, IL-5 and IL-13) responses to recall allergen challenges issued at 3 wk after DC10 immunotherapy (Fig 1C). Taken together, this data confirmed the regulatory activities of the DC10 used in this study.

### Impact of DC10 on the anatomic reservoirs for OVA-specific IgG1-secreting B cells in asthmatic mice

**Fig 2:**
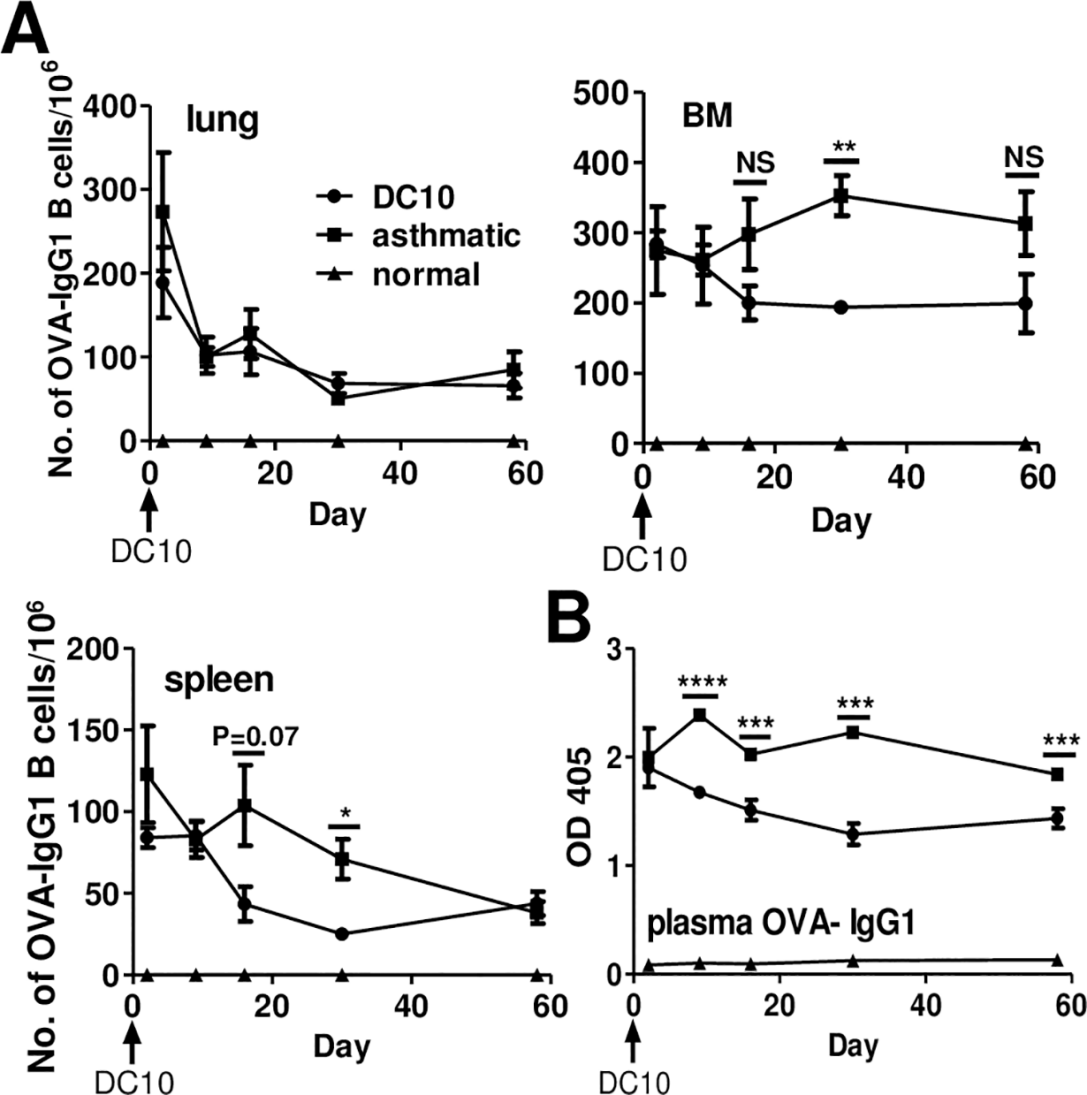
Assessment of the lungs, bone marrow (BM) and spleens of saline- or DC10-treated asthmatic mice as reservoirs for OVA-specific IgG1-secreting B cells. (**A)** Asthmatic BALB/c mice were given saline or DC10 treatments as indicated in Fig 1A; all mice were exposed to a recall allergen challenge 2 dy prior to sacrifice. Healthy, non-asthmatic mice were used as a negative control. At the indicated times (dy 2, 9, 16, 30, 58) we generated single cell suspensions from their lungs, BM and spleen, and assayed the numbers of OVA-specific IgG1-secreting B cells in 5 h ELISPOT assays, as noted in the Materials and Methods section. The data depicts the numbers of IgG1-secreting B cells per 1x10^6^ input cells, presented as the mean (±SEM) of 4 wells/sample. (**B)** Plasma was obtained from the animals in panel A and assayed for OVA-specific IgG1 antibody by ELISA with duplicate wells per sample, represented as the mean (±SEM). The data are representative of three experiments (for each, n = 4 or 5 for experimental mice, and 2 for normal control mice). The statistical analyses were performed using one-way ANOVA assays with Tukey’s *post-hoc* testing. **, ***, **** and NS signify p<0.01, 0.001, or 0.0001, and p>0.05 respectively.

We first wished to establish the natural history of allergen-specific IgG1-secreting B cells in untreated and DC10-treated asthmatic mice across time after asthma sensitization. As such, we used ELISPOT assays to assess the numbers of OVA-specific IgG1-secreting B cells in the lung parenchyma, bone marrow and spleens of asthmatic mice at varying times after asthma induction, beginning at the time of DC10 delivery; all mice were re-exposed to allergen via the airways 2 dy prior to sacrifice. We observed a rapid decline in the numbers of IgG1-secreting cells in the lungs of asthmatic mice after cessation of allergen challenge, and DC10 therapy had no further impact on this B cell pool, although a significant residual population of allergen-specific IgG1-secreting B cells remained in the lungs even 2 mo after cessation of allergen challenge (Fig 2A, upper left panel). In contrast, the numbers of IgG1 plasma cells remained more or less stable in the bone marrow (BM) of untreated asthmatic mice for the 8 wk duration of this study. DC10 therapy did have a significant impact on this bone marrow compartment, reducing the pool of OVA-specific IgG1-secreting bone marrow B cells by an additional 45+/-7.2% by day 30 (p≤0.01; Fig 2A). The splenic pool of these cells remained stable for ≈4 wk, but then declined, and here too the DC10 therapy further diminished the numbers of OVA-specific B cells (by 65+/-17.8%; p≤0.05) by day 30, although by dy 60 there were no differences in the numbers of residual IgG1-secreting B cells in the spleens of untreated and DC10-treated animals. At the end of this experiment all three compartments retained a significant reservoir of OVA-specific IgG1-secreting B cells, with the bone marrow carrying the bulk of this residual population (Fig 2A). Based on the total numbers of spleen and lung cells we recovered from our mice, and published values for the numbers of cells in the bone marrow of BALB/c mice [27], we calculate based on our two week data that the rank order for these organs as sources of allergen-specific IgG1-secreting B cells in our study was bone marrow (5x10^8^ cells) > spleen (1x10^8^ cells) > lungs (2x10^7^ cells). Given that we administered our DC10 intraperitoneally, we also assessed the prevalence of OVA-specific IgG1- and IgA-secreting B cells in that compartment–we were unable to detect any OVA-specific IgG1 or IgA-secreting B cells in peritoneal wash-out cell populations. Compared to saline-treated asthmatic mice, DC10 therapy also significantly reduced the plasma levels of OVA-specific IgG1 (Fig 2B) over the duration of this study, and we observed similar effects on the plasma levels of OVA-specific IgE and IgA in our mice (S2 Fig).

### DC10 can directly suppress allergen-specific B cell IgG1-secretion

**Fig 3:**
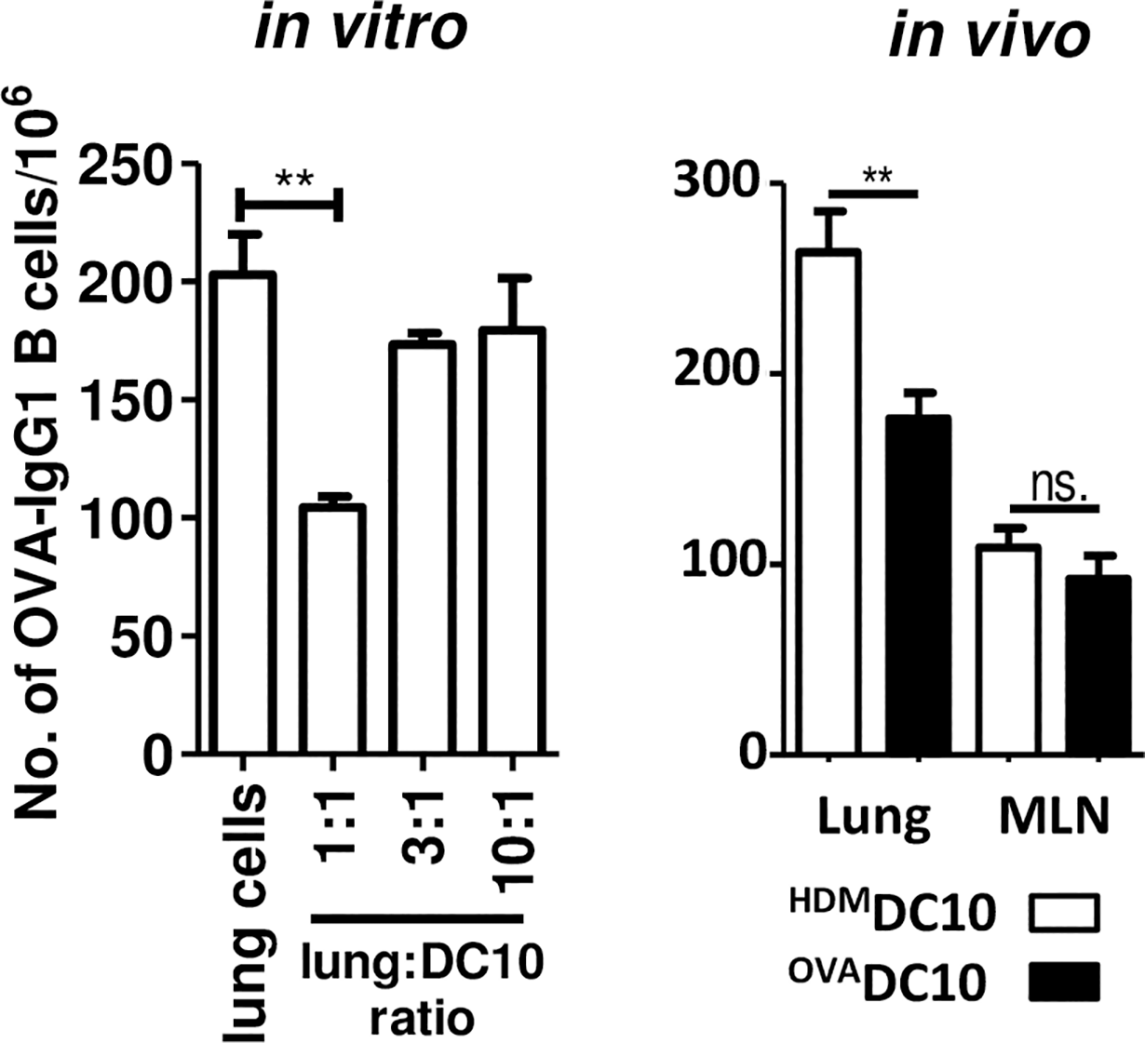
Allergen-loaded DC10 suppress IgG1 secretion by OVA-specific plasma cells both *in vitro* and *in vivo*. **(left panel)** Single cell suspensions were generated from the lungs of asthmatic BALB/c mice at 2 weeks after asthma induction and co-cultured with varying numbers of OVA-pulsed DC10 (none, or at lung cell:DC10 ratios of 1:1, 3:1, or 10:1) for 24 h before being transferred into ELISPOT plates for assay of IgG1 secretion. (**right panel)** DC10 were also injected transtracheally into the airways of asthmatic mice, and 2 dy later the mice were sacrificed and single cell suspensions generated from their lungs and lung-draining (mediastinal) lymph nodes (MLN). Aliquots of the lung single cells suspensions were added directly into the ELISPOT plates for 5 h assessments of OVA-specific IgG1 secretion, as in the left panel. DC10 suppressed IgG1 secretion *in vitro* in a cell number-dependent fashion. They also suppressed this B cell response within the lung tissues, but not the MLN, of asthmatic mice, as determined two days after DC10 delivery into the airways. The data is presented as the mean number of antibody-secreting cells/10^6^ input cells (±SEM) in 4 wells/sample, with each experiment being repeated 3 times (n = 4 mice/experiment). Statistical analyses were performed using *t*-test and one-way ANOVA assays with Tukey’s *post-hoc* testing. ** and NS signify p<0.05 and >0.05, respectively.

We wished to know which regulatory cell population is the primary driver of DC10-induced suppression of the allergen-specific IgG1 response. We first examined whether DC10 that had been pulsed overnight with OVA and then washed three times before use could directly suppress B cell responses *in vitro*, but also whether they could also do so *in vivo*. Thus, we co-cultured single cell suspensions from the lungs of asthmatic mice (as a source of allergen-specific antibody-secreting B cells) with the indicated numbers of DC10 for 24 h, and then assessed the numbers of OVA-specific IgG1-secreting B cells using 5 h ELISPOT assays. We found that the addition of DC10 reduced the numbers of IgG1-secreting B cells in a concentration-dependent fashion in this *in vitro* system (Fig 3, left panel). It has been reported previously that DC that are instilled into the airways are able to migrate across the respiratory epithelium into the lung tissues, and thereby to participate in local immune responses [28,29]. Thus, we also asked whether delivery of DC10 directly into the airway could impact lung and lung-draining (i.e., mediastinal) lymph node (MLN) IgG1-secreting B cells *in situ*. We injected 1x10^6^ specific (OVA) or irrelevant (house dust mite; HDM) allergen-loaded DC10 transtracheally into OVA-asthmatic mice, allowed these cells two days to gain access to and interact with local B cells, and then harvested the lungs and MLN for ELISPOT assay of OVA-specific IgG1 expression. We found that the OVA-presenting DC10 suppressed IgG1 expression by the lung, but not MLN B cells, reducing the lung response by ≈33+/-9.7% relative to the HDM-presenting DC10 (Fig 3, right panel). We also assessed the impact of DC10 on OVA-specific IgA secretion, and found that they similarly suppressed the IgA response (S3 Fig). This confirmed the allergen-specificity of DC10 suppression of the plasma cell response, but also that these DC10 can suppress lung plasma cell responses *in vivo*.

**Fig 4:**
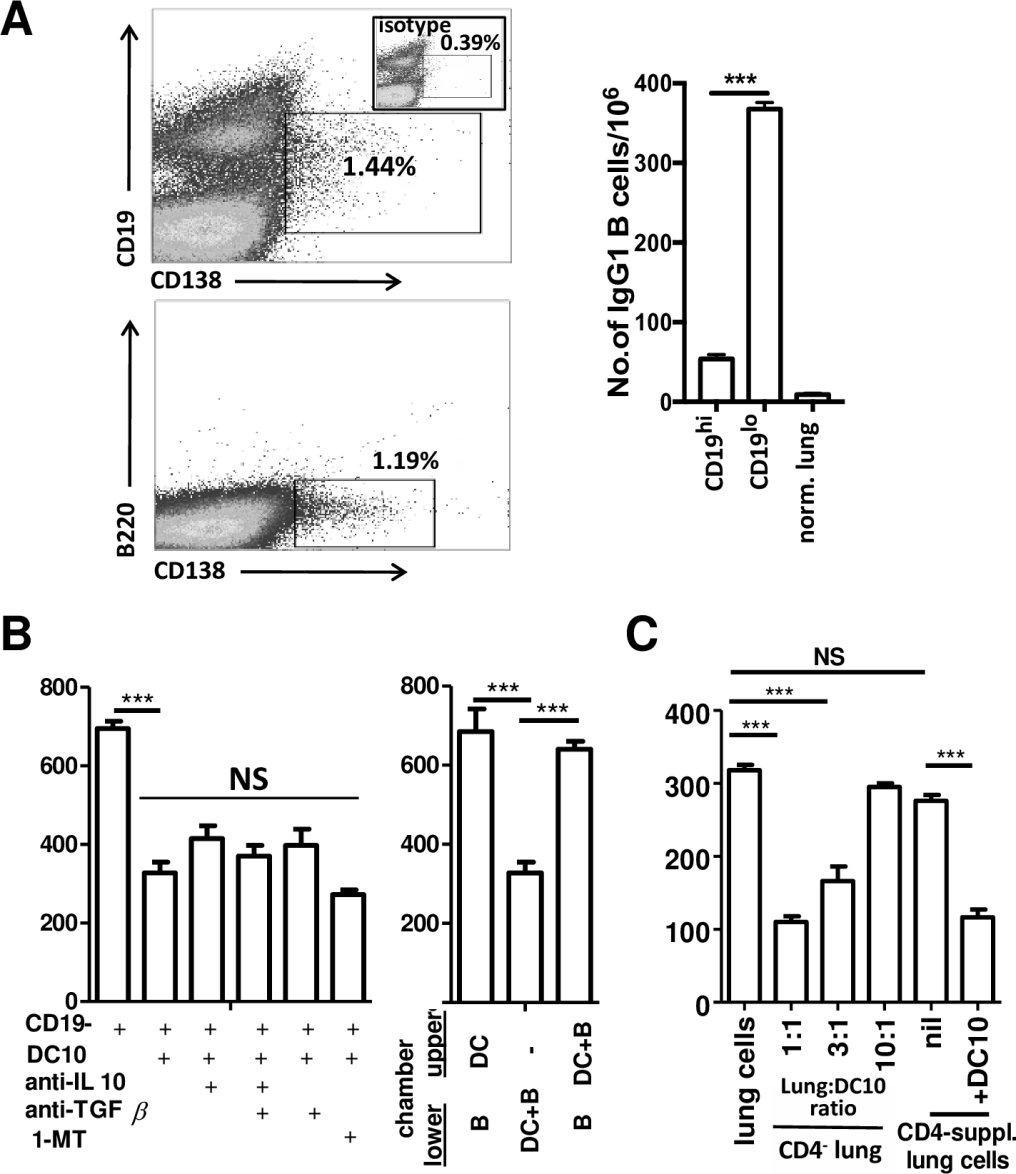
DC10 suppression of the IgG1 B cell response does not dependent on secreted mediators or T helper cells, and is contact-dependent. (**A, left panel)** Enzymatically-dispersed asthmatic lung single cell suspensions generated as in Fig 3 were analyzed by FACS for expression of CD19, CD138 and B220 using specific fluorochrome-labelled antibodies. The analysis was restricted to lymphocytes as assessed by forward- and side-scatter characteristics, and the gates set using irrelevant isotype control antibodies (eg, inset in the top panel). **(right panel)** The lung cells were magnetically-sorted using CD19-specific paramagnetic beads into CD19^+^ and CD19^-/lo^ populations, each of which was assessed directly in 5 h ELISPOT assays for OVA-specific IgG1-secreting cells. As expected, the vast majority of IgG1 secretion was associated with the CD19^-/lo^ population; unfractionated healthy lung single cell suspensions were also assayed for IgG1-secreting cells as a negative control. (**B, left panel)** DC10 were cultured in standard tissue-culture plates with asthmatic CD19-depleted lung cells (1:1 lung cell:DC10 ratio) either as is, or in the presence of anti-IL-10 and/or anti-TGFβ antibodies or the indoleamine-2,3-dioxygenase inhibitor 1-methyltransferase (1-MT) for 24 h and then transferred into ELISPOT plate for 5 h to assess for residual IgG1 secretion as above. None of these treatments significantly impacted DC10-dependent suppression of IgG1 secretion (p>0.05). (**B, right panel)** The need for direct B cell:DC10 contact was assessed using transwell approaches, with the cells either in different (i.e., upper versus lower) transwell chambers or in the same chamber for 24 h, and then cells on the bottom chamber were transferred into ELISPOT plates for 5 h. Whether direct DC10-B cell contact in the upper chamber induced the secretion of a soluble mediator(s) that could suppress IgG1 secretion by B cells in the lower chamber was also assessed. DC10-mediated suppression of IgG1 secretion was contact dependent, and not mediated by secreted mediators. **(C)** CD4^+^ T cell-depleted lung cells of asthmatic BALB/c mice (CD4^-^ lung cells) were co-cultured with varying numbers of OVA-presenting DC10 for 24 h, and then transferred into ELISPOT plates and assayed for B cell IgG1 production as in panel B. We also examined the impact of adding purified asthmatic lung CD4^+^ T cells back into these CD^+^ T cell-depleted lung cells in numbers equivalent to those seen in the asthmatic lung (i.e., 19%) and assessed the impact of DC10 co-culture with those cells. The CD4^+^ T cell-depleted lung cells contained as many OVA-specific IgG1-producing B cells as did the lung cells reconstituted with purified CD4^+^ T cells, indicating that the CD4^+^ T helper cells were dispensable for IgG1 production, and had no impact on DC10 suppression of this response. The data is presented as the mean number of antibody-secreting cells/10^6^ input cells (±SEM) in 4 wells/sample, and is representative of three independent experiments (n = 4/group). Statistical analyses were performed using *t*-tests (paired) and one-way ANOVA assays with Tukey’s *post-hoc* testing. *** and NS signify p<0.001 or p>0.05, respectively.

Given that mouse B cells lose expression of CD19 as they mature into plasma cells [30], we asked whether the pulmonary CD19^-/lo^ cell pool held the IgG1 secretory activity in our asthmatic mice. As expected, <10% of IgG1 secretion emanated from the CD19^+^ B cell pool, with the CD19^-/lo^ cells being responsible for the vast majority of the IgG1 response (Fig 4A; p≤0.001). We next turned to examining the cellular mechanisms regulating DC10 suppression of the B cell responses, co-culturing DC10 with CD19^-/lo^ lung cells, but also adding in neutralizing anti-IL-10 or TGFβ antibodies, or the indoleamine-2,3-dioxygenase (IDO) antagonist 1-methyltryptophan (1-MT). When cultured at a 1:1 ratio, DC10 down-regulated the CD19^-/lo^ B cell OVA-specific IgG1 response by ≈53% (p≤0.0001 versus untreated CD19^-/lo^ B cells). Although we know that DC10 suppress T cell responses through their secretion of IL-10 (15) and expression of indoleamine-2,3-dioxygenase (IDO)[16], neutralization of IL10, TGFß, or both, or inhibiting IDO with 1-methyl transferase (1-MT) in these cultures did not discernibly affected plasma cell suppression by the DC10 (Fig 4B). Thus, we next queried by use of transwell approaches whether DC10 suppression of the B cell IgG1 response called for direct DC10-B cell contact. We observed no suppression when the two populations were separated by a permeable membrane, but saw strong suppression when the DC10 and plasma cells were in direct contact with one another. To further assess the nature of the cell-cell contact that was responsible for DC10 suppression of the B cell response, we asked whether direct contact between DC10 and the plasma cells might induce secretion of a mediator with plasma cell suppressive activities. Thus, we cultured DC10 and lung cells together in the upper chamber of our transwells and placed putative target B cells in the lower chambers, and then assessed the numbers of IgG1-secreting plasma cells in the lower chambers 24 h later. We observed no suppression of IgG1 secretion by these B cells under these conditions (Fig 4B), clearly indicating that DC10 suppression of the plasma cell response in this *in vitro* model was fully contact-dependent.

Since we know that DC10 can suppress the functions of Th2 cells [16] and that helper T cells are important to the development of IgE and IgG1 responses in allergic disease [31], we queried whether a DC10-driven attrition of the helper T cell pool could be a factor that influenced DC10-induced B cell suppression. Thus, we used positive-selection magnetic sorting to deplete CD4^+^ T cells from asthmatic mouse lung cells, and then either used these cells as is (≈3% residual CD4+ cells; data not shown) or added back the sorted CD4^+^ cells to bring the final CD4+ T cell concentration up to the levels seen in unfractionated asthmatic lungs (19%) before assessing the impact of DC10 treatment on the IgG1 secretion responses in these populations (Fig 4C). The addition of the DC10 to the Th2 cell-depleted lung cells led to a DC10 concentration-dependent suppression of the B cell response, and supplementing these cells with asthmatic CD4^+^ T cells did not discernibly affect DC10 suppression of the B cell response. These data indicate that a lack of Th2 helper activity did not significantly contribute to the ability of DC10 to diminish allergen-specific IgG1 secretion.

### Direct DC10 suppression of IgG1-secreting B cells, but not Th2 cell proliferation, is dependent on DC10 presentation of intact allergen on their cell surface

**Fig 5:**
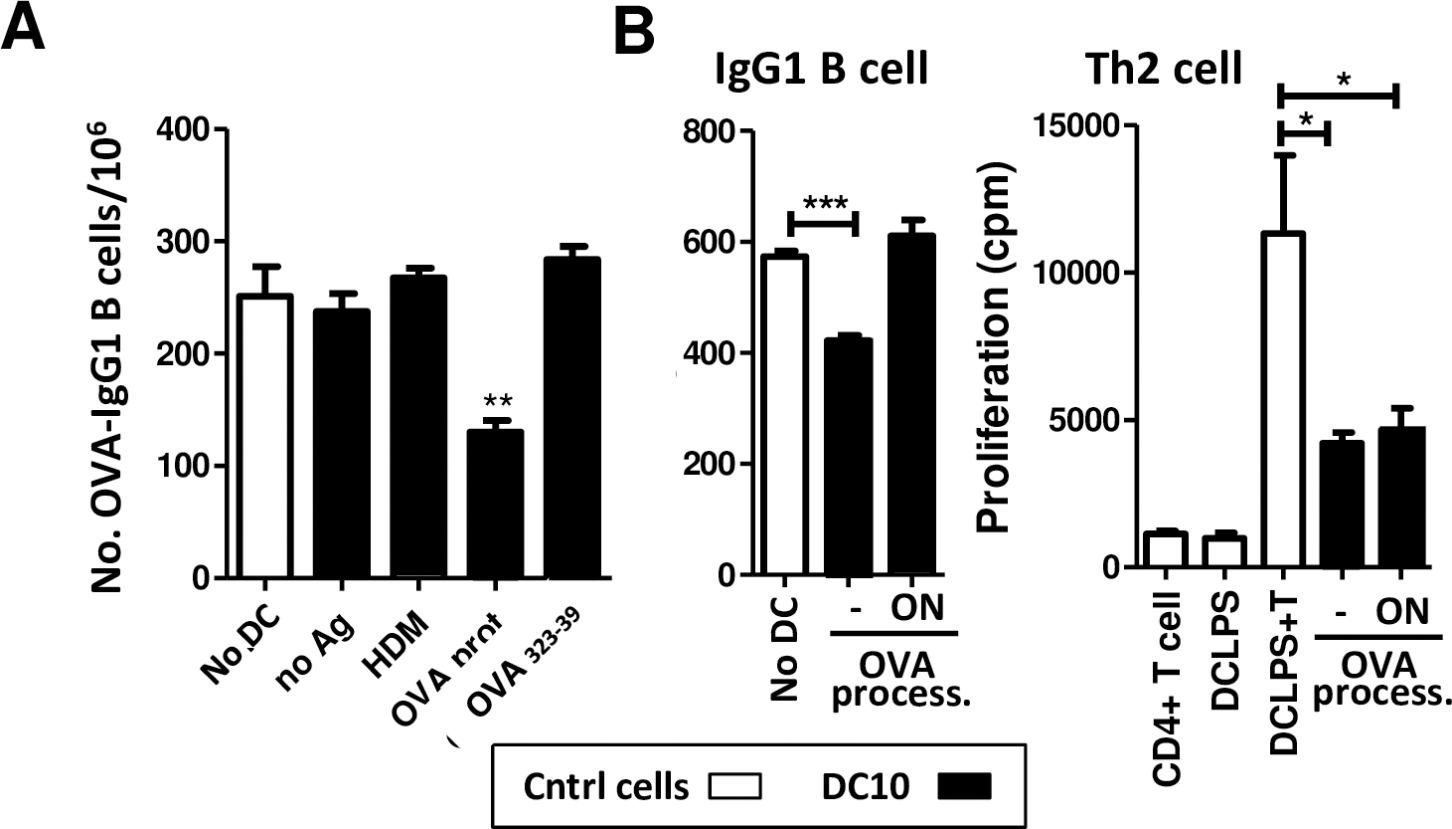
DC10 suppression of IgG1 secretion by OVA-specific B cells is antigen-specific, but requires DC presentation of intact allergen on their cell surface. (**A)** To assess the antigen specificity of DC10-dependent IgG1 plasma cell suppression, we pulsed DC10 with no antigen (no Ag), an irrelevant allergen (house dust mite; HDM), intact OVA protein or OVA peptide_323-39_ and assessed their abilities to inhibit IgG1 secretion as above. The DC10 and lung cells were incubated for 24 h in 96-well U-bottom plates, and then transferred into ELISPOT plates for 5 h. **(B)** To confirm that DC10 presentation of intact cell surface-associated allergen to B cells (versus processed allergen) is required for suppression of IgG1 production, we pulsed DC10 with OVA for 2 h and washed them, and then either used them without allowing any further time for antigen processing (- OVA process.), or we returned them to the incubator overnight to allow full allergen processing (ON OVA process.). We then assessed the abilities of both populations to suppress either OVA-specific IgG1 secretion as above (left panel) or, as a control, immunostimulatory DC (DC-LPS)-activated asthmatic CD4^+^ T cell proliferative responses (right panel) by use of standard ^3^H-thymidine uptake assays. For suppression of OVA-specific IgG1 plasma cells, the cells were co-incubated for 24 h in 96 U-bottom plate before transfer into ELISPOT plates for 5 h. Control wells for the T cell suppression assay included lung CD4^+^ T cells alone, DC-LPS alone, and DC-LPS and T cells, but without DC10. The DC10 that had been given time to process their allergen were unable to suppress IgG1 secretion but, as expected, were able to suppress the T cell response. This data is representative of three independent experiments (n = 4/group). Statistical analyses were performed using one-way ANOVA assays with Tukey’s *post-hoc* testing. *, ** and *** signify p<0.05, 0.01 and 0.001, respectively.

We next examined the conditions under which DC10 can suppress the B cell IgG1 response, co-culturing CD19^-/lo^ asthmatic mouse lung cells with DC10 that had not been exposed to any allergen, or ones that had been loaded with either irrelevant (house dust mite; HDM) or cognate (OVA) allergen. We found that DC10 that had not been exposed to any allergen, or those that presented HDM allergen were ineffective in suppressing the OVA-specific B cell IgG1 response, while DC10 that had been pulsed with OVA protein and washed three times were able to suppress this response. Interestingly, DC10 that had been loaded with MHCII-restricted OVA peptide_323-339_ in place of intact OVA did not discernibly inhibit the B cell response (Fig 5A).

It has been reported that DC that have phagocytosed antigens [18,19] can in turn pass those antigens, in an intact form, directly to B cells and thereby induce robust B cell responses [19], so we next assessed whether this could be a mechanism for DC10 suppression of the IgG1 response in our model. Thus, we pulsed our DC10 with intact OVA protein and washed them, and then either used them immediately after washing, or returned the cells to culture overnight to allow them to phagocytose and process their allergen load. The DC10 that were used immediately after washing did suppress the B cell IgG1 response, while the cells that were allowed to process their OVA had no impact on IgG1 secretion, although both populations of DC10 were fully capable of suppressing asthmatic lung Th2 cell proliferative responses to allergen-presenting immunostimulatory DC-LPS (Fig 5B).

**Fig 6:**
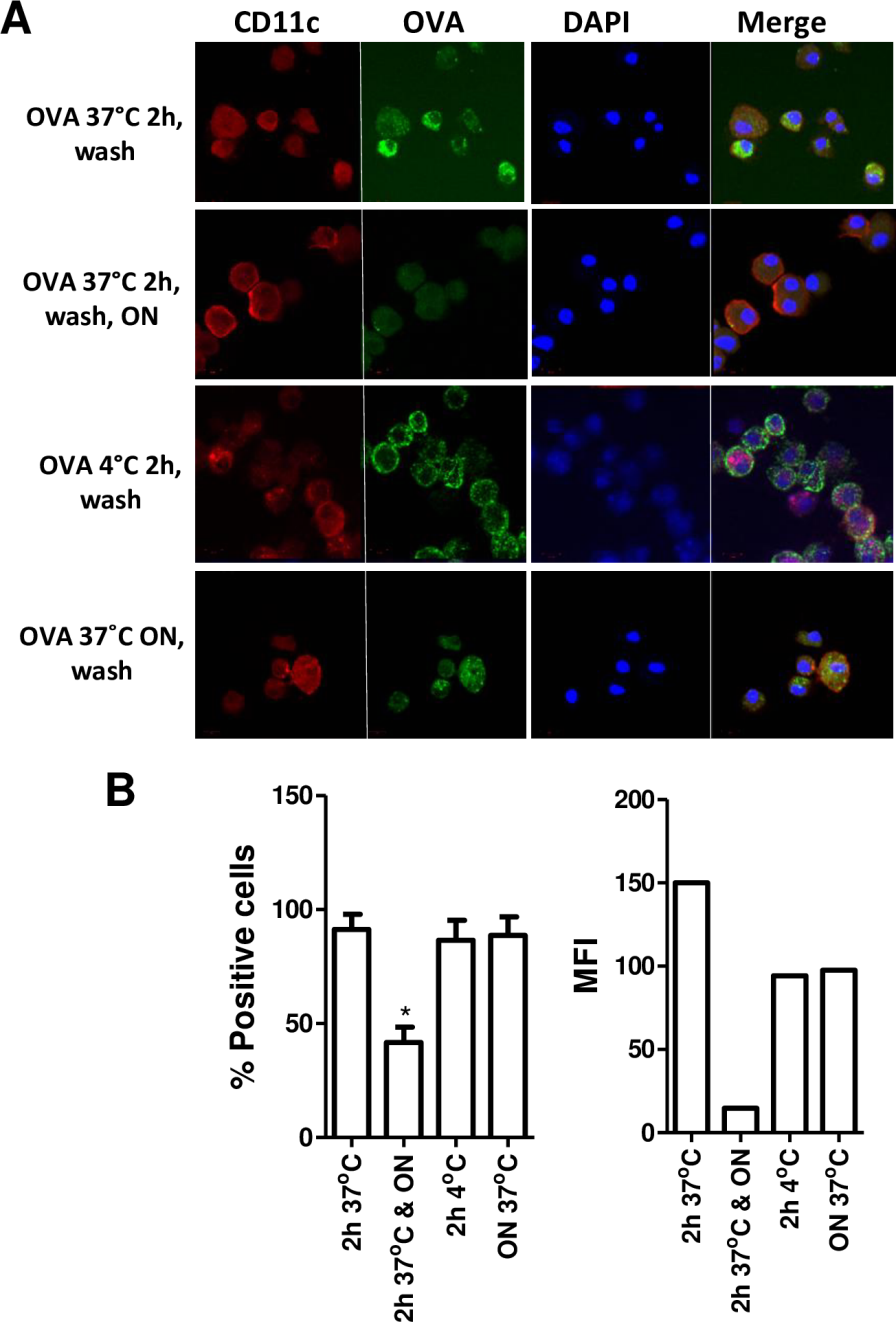
Confocal imaging and FACS analysis of allergen processing by DC10. DC10 were pulsed with FITC-labeled OVA for 2 at either 4 (OVA 4°C 2 h, wash) or 37°C (OVA 37°C 2 h, wash), and washed, and then either used as is or allowed to process their allergen overnight (OVA 37°C 2 h, wash, ON) as in Fig 5. Control DC10 were pulsed with FITC-OVA overnight at 37°C before washing (OVA 37°C ON, wash) and subsequent analysis. **(A)** The DC10 were then incubated for 10 min with 0.5 μg Fc block and stained with PE-anti-mouse CD11c (or isotype control antibodies), then fixed with 4% paraformaldehyde before being applied to cytocentrifuge slides and stained with the nuclear dye DAPI. Zeiss LSM 700 confocal microscope images of CD11c, FITC-OVA and DAPI-stained cells, and merged images of all three stains are presented for each treatment. **(B)** The fixed cells were also analyzed by FACS, with cell gating based on isotype control antibody staining; the FACS data is presented as **(left panel)** the percent of the cells staining positively for FITC-OVA and **(right panel)** the mean fluorescence intensity of FITC-OVA staining. Approximately 40% of the DC10 that had been washed and allowed to process their residual FITC-OVA displayed close to background levels of FITC staining. This data is representative of three independent experiments. Statistical analyses were performed using one-way ANOVA assays with Tukey’s *post-hoc* testing.

We also assessed the cell surface levels of intact OVA that were associated with DC10. To do this, the cells were either exposed to FITC-OVA for 2h at 4 (2h 4°C) or 37°C (2h 37°C), or overnight at 37°C (ON 37°C), and then washed and examined them immediately by confocal microscopy and FACS (Fig 6). Another group of cells was exposed to FITC OVA for 2 h at 37°C and then washed and placed into fresh medium overnight at 37°C to allow the cells to phagocytose and process the OVA (2h 37°C & ON) before our analysis. We found that DC10 that had been exposed to OVA but not given time to clear this allergen (OVA 37°C h, wash; OVA 4°C 2 h, wash; OVA 37°C ON, wash) each had substantial amounts of intact OVA on their cell surface. On the other hand, the DC10 that had been pulsed with OVA for 2 h, washed and then allowed to process the allergen overnight (OVA 37°C 2h, wash, ON) had little if any residual intact cell-surface FITC-OVA, although the confocal and FACS analyses were able to discern low levels of signal from the phagocytosed OVA (Fig 6A and 6B). Taken together, this data confirms that DC10 that are carrying intact allergen on their cell surface are able to suppress B cell responses, but that they lose this ability once they have processed their allergen.

### DC10-induced Treg do suppress the OVA-specific B cell IgG1 response

**Fig 7:**
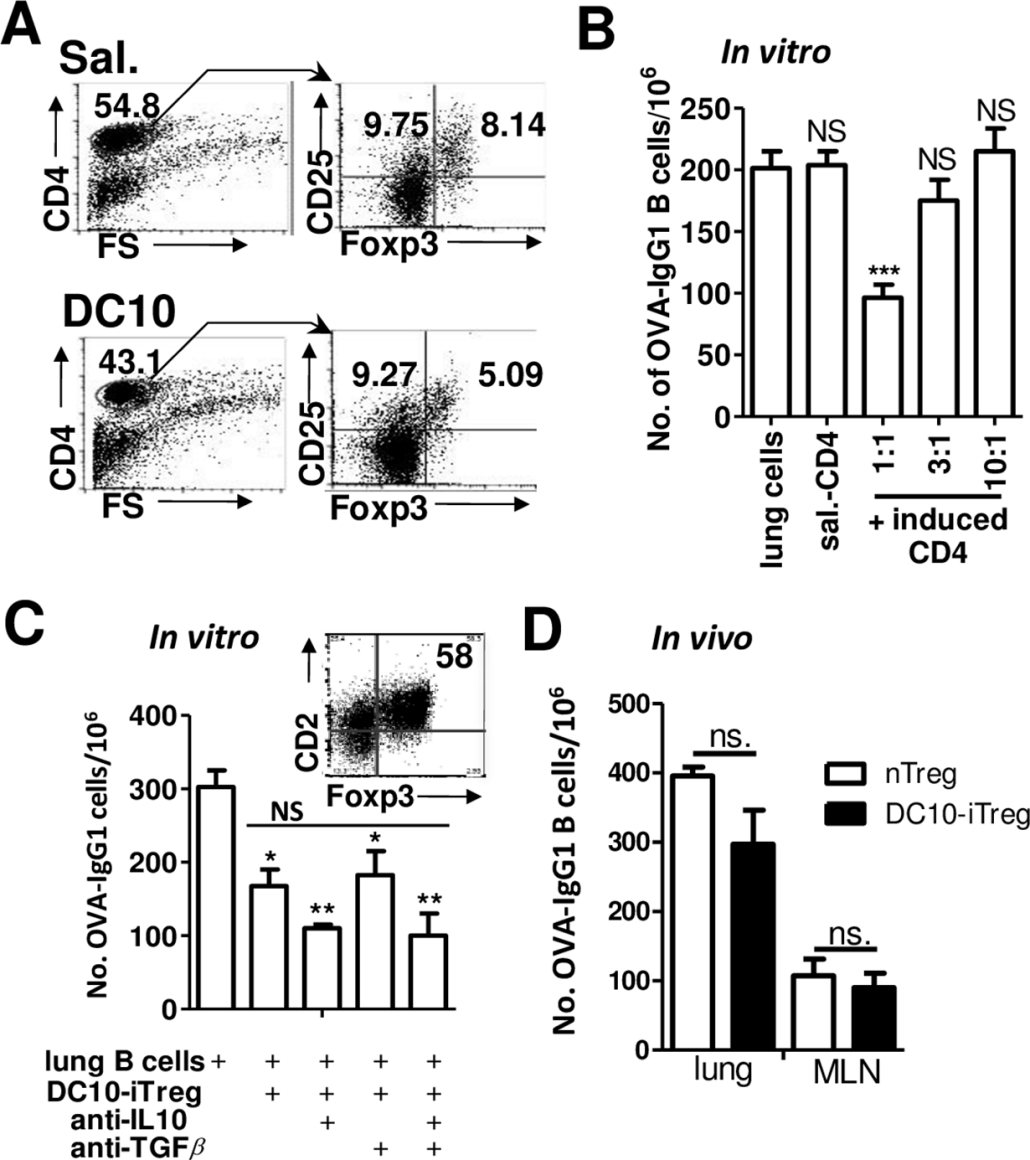
DC10-induced CD4^+^ regulatory T cell suppression of OVA-specific IgG1-secreting B cell responses. CD4^+^ lung cells magnetically sorted from lung single cell suspensions generated 3 wk after treatment of asthmatic mice with saline (Sal.) or DC10 were **(A)** analyzed for expression of the regulatory T cell (Treg) markers CD25 and Foxp3 by FACS. (**B)** These CD4^+^ T cells, known to be rich in activated allergen-specific Treg, were co-cultured with asthmatic lung cells for 24 h at the indicated B cell:CD4^+^ T cell ratios, and then they were transferred into ELISPOT plates for 5 h to assess their impact on OVA-specific IgG1 B cells. Positive and negative control wells contained lung cells alone as a source of IgG1-secreting B cells, and lung cells that had been co-cultured with CD4^+^ T cells from saline-treated asthmatic animals (sal. CD4), respectively. **(C)** We also magnetically-sorted CD25^+^Foxp3^+^ Treg from the lungs of DC10-treated asthmatic (DC10-iTreg; inset, 58% CD25^+^Foxp3^+^ cells) mice and assessed their abilities to reduce the IgG1 response as in panel B, setting up the cells at a 1:1 iTreg:lung cell ratio, but also assessing the impact of neutralizing anti-IL-10 and/or -TGFβ antibodies as in Fig 4. The purified DC10-induced Treg suppressed IgG1 secretion, but neutralization of IL-10 and/or TGFβ had no significant impact on this suppression (p>0.05). (**D)** We generated DC10-induced Treg *in vitro*, as noted in the Materials and Methods, and sorted CD25^+^ natural Treg (nTreg) from the lungs and spleens of normal mice, and then injected 1x10^6^ of these cell transtracheally into asthmatic mice. Two days later we generated single cell suspensions of lung and MLN cells from the treated mice and assessed these in 5 h ELISPOT assays for OVA-specific IgG1-secreting B cells as in Fig 3. Each bar in the graphs represents the mean (±SEM) of 4 wells. These results are representative of three independent experiments, with n = 3 or 4/group. Statistical analyses were performed using one-way ANOVA assays with Tukey’s *post-hoc* testing (panels B & C) or *t*-testing (panel D). *, **, *** and NS signify p<0.05, 0.01, 0.001 and p>0.05, respectively.

As noted above, we had previously documented that DC10 therapy in asthmatic mice induces Th2 cells to differentiate into CD25^+^Foxp3^+^ Treg, with maximal Treg induction and activation occurring at 3 wk after DC10 delivery [14]. Thus, we next asked whether DC10-induced Treg can suppress IgG1 expression among allergen-specific B cells. We had previously reported that while the numbers of CD25^+^Foxp3^+^ cells in the lungs of asthmatic mice do not increase as a consequence of DC10 therapy, DC10 therapy does lead to their activation such that pulmonary CD25^+^Foxp3^+^ cells from saline-treated asthmatic mice are only poorly regulatory, while the analogous activated cells from DC10-treated asthmatic mice are strongly regulatory [14]. We magnetically sorted CD4^+^ T cells from the lungs of saline- or DC10-treated asthmatic mice at week 3 after treatment, co-cultured these sorted T cells with CD4^+^ T cell-depleted lung cells, as a source of B cells, and then assessed IgG1 secretion. The sorted CD4^+^ cells from the lungs of the saline- or DC10-treated asthmatic mice contained 8.1% and 5.1% CD25^+^Foxp3^+^ T cells, respectively (Fig 7A). The CD4^+^ T cells from DC10-treated asthmatic mice suppressed OVA-IgG1 B cell secretion in a concentration-dependent manner, with a 52.2+/-8.7% suppression being observed at a 1:1 ratio, while the CD4^+^ lung cells from saline-treated asthmatic mice had no such activities (Fig 7B). We confirmed the identity of the cells carrying this activity by use of magnetically sorted CD4^+^CD25^+^ regulatory T cells (58% CD25^+^Foxp3^+^ T cells; inset, Fig 7C), also from the lungs of DC10-treated asthmatic mice, which dampened the OVA-IgG1 B cell response by 44.6+/-12.2%. We had reported previously that IL-10 secretion by DC10 was critical to their regulation of Th2 cells [15], so we also assessed the impact on IgG1 secretion of neutralizing IL-10, TGF-β or both in this *in vitro* system; neither IL-10 or TGFβ contributed discernibly to iTreg suppression of the IgG1 response (Fig 7C). Lastly, we asked whether DC10-induced Tregs would suppress OVA-IgG1 B cells *in vivo*, in a manner similar to that we used for our *in vivo* DC10 assay (Fig 3), injecting DC10-iTreg or, as a control, CD25^+^Foxp3^+^ Treg from normal mice transtracheally into asthmatic BALB/c mice. Two days later we harvested the lungs and MLN from the treated mice and assayed these for OVA-specific IgG1-secreting B cells by ELISPOT. We did not detect any discernible effect of the DC10-iTreg (or nTreg) on OVA-specific IgG1 secretion by B cells from the lungs or lung-draining lymph nodes in this assay (Fig 7D). We also assessed whether DC10-induced Treg could suppress IgA responses *in vivo*, but found that as with IgG1, they had no impact on the IgA response in this assay (S3 Fig). We reported previously that DC10-induced Treg can readily suppress OVA-specific IgE and IgG1 responses in asthmatic mice following i.v. injection, which suggests that the failure of the Treg to impact the IgG1 response herein could be because, unlike DC [28,29], T cells may well not be able to migrate from the airways into the lung parenchyma.

## Discussion

It has been reported previously that DC10 treatments reverse the disease phenotype in OVA and house dust mite models of asthma, wherein they suppress the allergen-specific T cell response [7,8,9,16], inducing Th2 cells to differentiate into CD4^+^CD25^+^Foxp3^+^ Treg [14]. The impact of DC10 treatment on serum allergen-specific IgE/IgG1 levels lags somewhat behind their effects on the airway Th2 recall responses to allergen challenge or on airway hyperresponsiveness (AHR), but nevertheless the levels of IgE and IgG1 in DC10-treated mice approach background within ≈4 months of a single treatment [16]. Reductions in IgE levels could with time reduce mast cell responsiveness to allergen triggering [5], while reductions in IgG1 levels would decrease IgG1-allergen immune complex formation, and thereby downstream activation of Fc-gamma RIII- and Fc-gamma RIV-bearing cells such as mast cells, basophils and eosinophils that contribute to the pathogenesis of asthma [4]. However, the mechanisms by which DC10 affect the B cell response(s) had not been defined. It would seem clear from the present study that while DC10 that carry intact allergen on their cell surface can directly suppress allergen-specific IgG1 antibody secretion, likely due to combined inhibitory receptor (e.g., PD-L1) and allergen signaling, once they have fully processed their allergen their plasma cell regulatory activities are largely realized indirectly through alternate regulatory cells they induce (e.g., Treg). The cell surface allergen-dependent suppression of IgG1 secretion we observed would have occurred within the 24 h time-frame of the initial DC10-plasma cell interactions (i.e., prior to their transfer into ELISPOT plates), inasmuch as DC10 that were given 24 h to process their allergen had only background levels residual allergen on their cell surface, as determined by confocal microscopy and FACS, and were no longer capable of suppressing the IgG1 response. This also provided confirmation that residual unprocessed allergen associated with DC10 could not have dampened the ELISPOT assay signals simply by virtue of having adsorbed the IgG1 secreted within the ELISPOT plates.

We previously reported that substantial numbers of IgA-secreting B cells can be detected in the lungs of asthmatic mice [3], but we did not assess whether allergen-specific B cells take up residence in that compartment for extended periods of time, or whether they eventually involute *in situ* or emigrate after cessation of allergen exposure. Short-lived plasma cells, which express CD138 and lower levels of CD19 [32], reportedly disappear quickly on cessation of antigen exposure [32], and this fits well with the observed presence of CD19^-/lo^CD138^+^ plasma cells in the lungs of our asthmatic mice, but also with the rapid decline in IgG1-secreting cells we observed in this study. On the other hand, long-lived plasma cells, which express CD138 but not CD19, survive for prolonged periods of time in the spleen and bone marrow, where local stromal cells supply the necessary survival factors (eg, APRIL, BAFF, IL-6)[32]. Our observation of IgG1-secreting B cells that survived in large numbers in the bone marrow is consistent with prior reports that this compartment is a major depot for long-lived plasma cells, which are reportedly the source of 80% of serum antibody [33]. We did not assess whether the rapidly lost IgG1-secreting B cells in these latter compartments were short-lived plasma cells or long-lived cells that were susceptible to DC10-induced suppression, by whatever means. However, we did find a small but discrete population of IgG1-secreting B cells that survived in the lungs for a prolonged period, even in the absence of allergen exposure, but we did not investigate the nature of these cells so we cannot comment on whether they may have been a novel population of long-lived pulmonary plasma cells. While we did not observe a straightforward correlation between OVA-specific IgG1-secreting cell numbers in the bone marrow and the plasma levels of this antibody, the general trends for these two parameters were not dissimilar (i.e., an early peak with a decline thereafter). It has been reported that long-lived plasma cells are resistant to immunosuppression, at least in terms of susceptibility to cyclophosphamide, anti-CD20 antibodies [34] or X-irradiation [35], but our DC10 treatment did significantly suppress IgG1-secreting plasma cells in the bone marrow and spleens of our asthmatic mice within 2–3 wk of treatment. Nevertheless, IgG1-secreting B cells maintained a marked presence in the bone marrow, with a more modest presence in the spleen and lungs at the 8 wk time-point. We have reported elsewhere that within ≈4 months of DC10 treatment the circulating levels of allergen-specific IgE and IgG1 in asthmatic mice achieve those seen in normal, non-asthmatic animals, while IgE and IgG1 levels remain relatively elevated in untreated control asthmatic mice [16]. Thus, while a subpopulation of otherwise long-lived plasma cells may be somewhat resistant to DC10 treatment, this resistance must eventually break down [16].

In principle, a number of cell populations could be central effectors of DC10 suppression of the IgG1 response, including the DC10 themselves, Th2 helper cells, removal of which could attenuate antibody production, and DC10-induced Treg [14] or endogenous pulmonary DC that we know to take on a regulatory phenotype after DC10 therapy (C. Li, W. Dawicki & JR Gordon, submitted for publication) [36]. Dendritic cells do express a number of markers or mediators that, in principle, allow them to interact directly with B cells. Thus immature, but not mature bone marrow-derived DC express a ST6Gal-I sialyltransferase-independent ligand for the inhibitory B cell receptor CD22 (i.e., a second CD22L), such that contact-dependent engagement with these immature DC can reduce anti-IgM-induced B cell proliferation [20]. DC10 express very low levels of CD40 and CD54 [16], while B cells express CD40L [37] and LFA-1 (reviewed in ref 20), their respective counter-receptors. We know that DC10 secretion of IL-10 is a critical element in their abilities to induce tolerance in asthma [15], such that we were initially surprised that DC10 inhibition of the B cell response was contact-dependent. Having said that, it has been suggested that IL-10 may have stimulatory, as opposed to inhibitory effects on B cells [38]. For example, IL-10 reportedly can foster IgE production by B cells [39] and it has been suggested that allergen-specific immunotherapy-induced IL-10 expression may induce B cell isotype switching, with increases in IgG4 expression [40] rather than overt suppression of the B cell response. In any case, the fact that we observed no suppression of IgG1 secretion *in vitro* by DC10 that were not carrying intact allergen on their cell surface suggests that neither mediators secreted by DC10, or any inhibitory receptors they may express on their cell surface [41], by themselves, would have contributed to DC10 suppression of the IgG1 response.

We previously reported that after i.p. delivery to asthmatic mice, DC10 migrate from that compartment and increasingly accumulate in the airways, lung parenchyma and lung-draining lymph nodes and spleens over 7 days [15]. We estimate based on the time required for DC10 to fully process their allergen (i.e., ≤24 h) that allergen-laden DC10 that are given i.p. could in large part only interact directly with cognate B cells within the peritoneal cavity, although we detected no OVA-specific IgA or IgG1-secreting B cells in the peritoneal cavities of OVA-asthmatic mice. The observation that DC10 have largely disappeared from treated animals at 3 wk post-treatment [15], when lung, splenic and bone marrow IgG1 B cell numbers and plasma antibody levels are continuing to decline, suggests that an alternate population or populations of regulatory cells would have to take over this role following the disappearance of the treatment DC10. We know that the CD25^+^Foxp3^+^ Treg in the lungs of DC10-treated asthmatic mice begin to display significant regulatory activities within 1 wk of DC10 therapy, and that they achieve maximal activities at ≈3 wk [14]. This is consistent with the time-frame for DC10 therapy-induced reductions in OVA-specific plasma IgG1 and IgE levels observed herein. That maximal suppression of the allergen-specific IgG1 response takes ≈4 mo to be realized in asthmatic mice after one DC10 treatment [16] clearly supports this proposal that other cells must fully take over regulation of the allergic B cell response in DC10 recipients. Our observation that allergen-laden DC10 that are delivered into the airways can suppress lung parenchymal B cells *in situ* indicates that, unlike the Treg we delivered into the airway, they must be able to gain direct access to these B cells in a relatively short period of time (e.g., less than the time required for loss of intact cell surface allergen). We know that DC10 that are delivered transtracheally are highly effective in inducing full asthma tolerance, including airway hyperresponsiveness and the eosinophilic Th2 phenotype [16]. This suggests that endogenous lung-resident DCreg, such as those induced by DC10 therapy (C. Li, W. Dawicki, H. Huang, X. Zhang and J.R. Gordon, submitted for publication), that acquire cell surface allergen *in situ* in the lungs as a consequence of subsequent allergen exposure, could potentially also directly suppress local IgG1-secreting cells. Thus, our evidence indicates that DC10 induce B cell tolerance through the Treg, and potentially also the endogenous DCreg, that they induce. We had reported previously that DC10-induced Treg can readily suppress OVA-specific IgE and IgG1 responses in asthmatic mice following i.v. injection, which suggests that the failure of the Treg to impact the IgG1 response herein after transtracheal delivery could be because, unlike DC [28,29], T cells may not be able to migrate efficiently from the airways into the lung parenchyma. It would be interesting to know whether DC10 also induce differentiation of regulatory B cells (Breg) [42] in a manner not unlike their conversion of Th2 cells into Treg [14], but also to know what role(s) such putative Breg might play in the regulation of allergic disease in our model.

In summary, we have shown that DC10 and DC10-induced regulatory T cells both impact the antigen-specific IgG1- and IgA-secreting B cell responses. Reductions in IgG1 levels would decrease IgG1-allergen immune complex formation, and thereby downstream activation of a variety of innate immune cells of direct relevance to asthma pathogenesis [4] while parallel reductions in IgE levels could reduce mast cell and basophil responsiveness to allergen triggering [5]. Overall, our data indicates that therapeutic application of tolerogenic dendritic cells in the clinic would bring disease relief both through directed attrition of the Th2 responses as well as allergen-specific plasma cell responses.

## Supporting information

S1 Fig Relationship between the numbers of ELISPOT assay input cells and the numbers of IgG1-secreting B cells detected. Assessment of the linearity of the relationship between the numbers of OVA-IgA B cells detected and the numbers of input cells in our ELISPOT assay (r^2^ = 0.89). Single cell suspensions were generated from the lungs of asthmatic BALB/c mice at 2 weeks after asthma induction. The cells were aliquotted into ELISPOT plates for 5 h, using 10^5^, 2x10^5^, or 5x10^5^/well. The data depicts the numbers of OVA-specific IgG1-secreting B cells/well, and are presented as the mean (±SEM) of 4 wells/sample.
(TIF)

S2 Fig Impact of DC10 treatment on plasma IgE and IgA levels in asthmatic mice. **(A)** Assessment of the plasma OVA-specific IgE and IgA levels in saline- or DC10-treated asthmatic mice across time after DC10 treatment, as was done for IgG1 in Fig 2B. **(B)** Assessment of OVA-specific IgG1, IgE, IgA levels in BAL fluid of saline- or DC10-treated asthmatic mice on week 3 after treatment. Each data point represents the mean (±SEM) of duplicate wells. This data is representative of three experiments (n = 4 or 5 for experimental mice, and 2 for normal control mice). * and *** signify P<0.05 and 0.001, respectively.
(TIF)

S3 Fig OVA-, but not irrelevant allergen-loaded DC10 suppress IgA secretion by OVA-specific B cells both *in vitro* and *in vivo*. **(A)** Asthmatic lung single cell suspensions were co-cultured either alone (Nil) or with OVA-pulsed DC10 (lung cell:DC10 ratio of 1:1) for 24 h, and then transferred into ELISPOT plate for 5 h to analyze OVA-specific IgA B cells, as in Fig 3. (**B)** OVA-loaded DC10 was also injected transtracheally into the airways of asthmatic mice, and 2 dy later single cell suspensions were generated from their lungs and lung-draining (mediastinal) lymph nodes (MLN). Aliquots of the cell suspensions were assayed for OVA-specific IgA-secreting B cells in 5 h ELISPOT assays, as in panel A. DC10 suppressed IgA secretion *in vitro*, as well as within the lung tissues, but not the MLN of asthmatic mice. We also assessed the impact of natural Treg and DC10-induced Treg on lung and MLN IgA secretion, as was done for IgG1 in Fig 7. Neither the nTreg or iTreg had any impact on the lung or MLN IgA secretion in this assay, although we have reported previously that passive transfer of DC10-iTreg can fully suppress the asthmatic OVA-specific IgE and IgG1 response over 4 wk. The data is presented as the mean number of antibody-secreting cells/10^6^ input cells (±SEM) in 4 wells/sample, with each experiment being repeated 3 times (n = 4 mice/experiment). Statistical analyses were performed using one-way ANOVA assays with Tukey’s *post-hoc* testing. ** and NS signify p<0.05 and >0.05, respectively.
(TIF)
