# Supplementary figures and images for: Contributions of direct versus indirect mechanisms for regulatory dendritic cell suppression of asthmatic allergen-specific IgG1 antibody responses
Source: PLoS One. 2018 Jan 2;13(1):e0190414. doi: 10.1371/journal.pone.0190414 (PMC5749789; doi:10.1371/journal.pone.0190414)

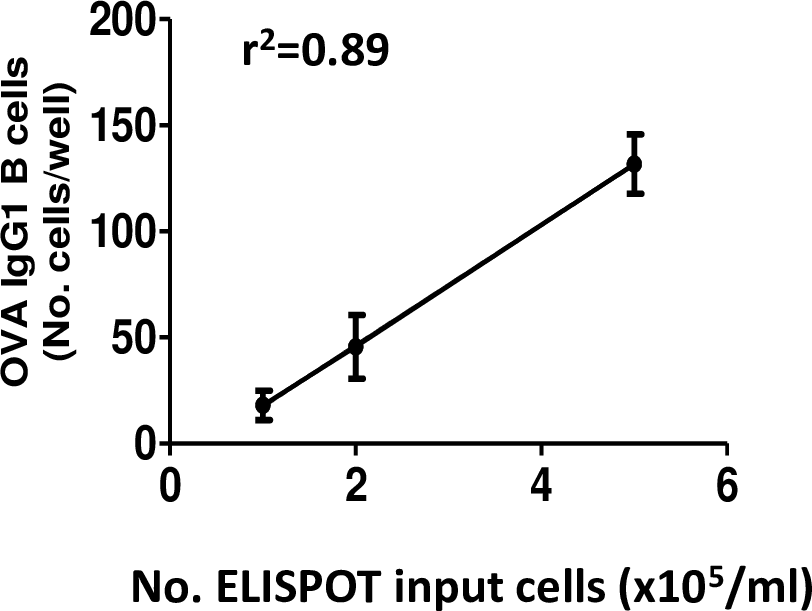

Supplement: S1 Fig — Assessment of the linearity of the relationship between the numbers of OVA-IgA B cells detected and the numbers of input cells in our ELISPOT assay (r2 = 0.89). Single cell suspensions were generated from the lungs of asthmatic BALB/c mice at 2 weeks after asthma induction. The cells were aliquotted into ELISPOT plates for 5 h, using 105, 2x10^5^, or 5x10^5^/well. The data depicts the numbers of OVA-specific IgG1-secreting B cells/well, and are presented as the mean (±SEM) of 4 wells/sample. (TIF) [file pone.0190414.s001.tif]

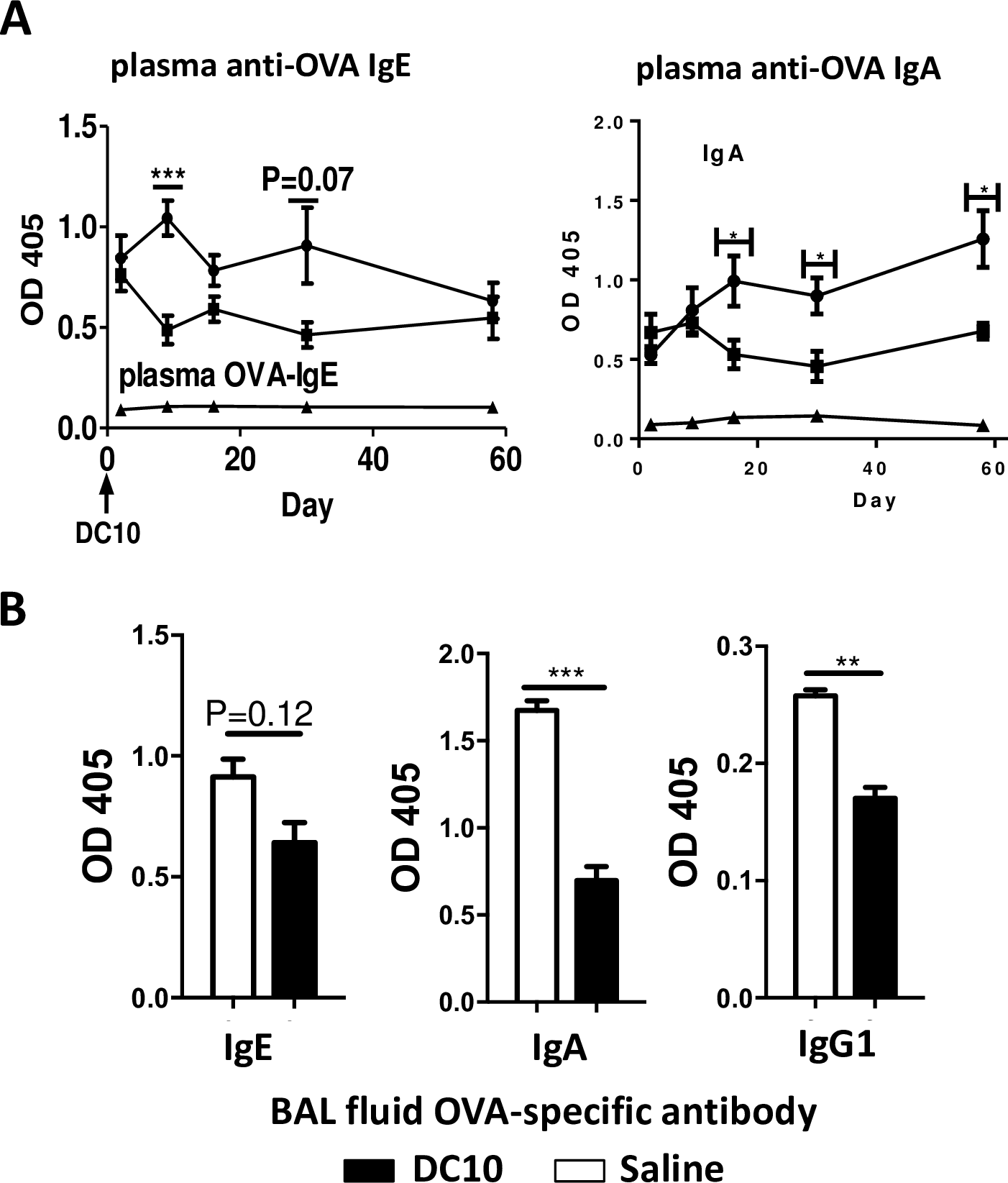

Supplement: S2 Fig — (A) Assessment of the plasma OVA-specific IgE and IgA levels in saline- or DC10-treated asthmatic mice across time after DC10 treatment, as was done for IgG1 in Fig 2B. (B) Assessment of OVA-specific IgG1, IgE, IgA levels in BAL fluid of saline- or DC10-treated asthmatic mice on week 3 after treatment. Each data point represents the mean (±SEM) of duplicate wells. This data is representative of three experiments (n = 4 or 5 for experimental mice, and 2 for normal control mice). * and *** signify P<0.05 and 0.001, respectively. (TIF) [file pone.0190414.s002.tif]

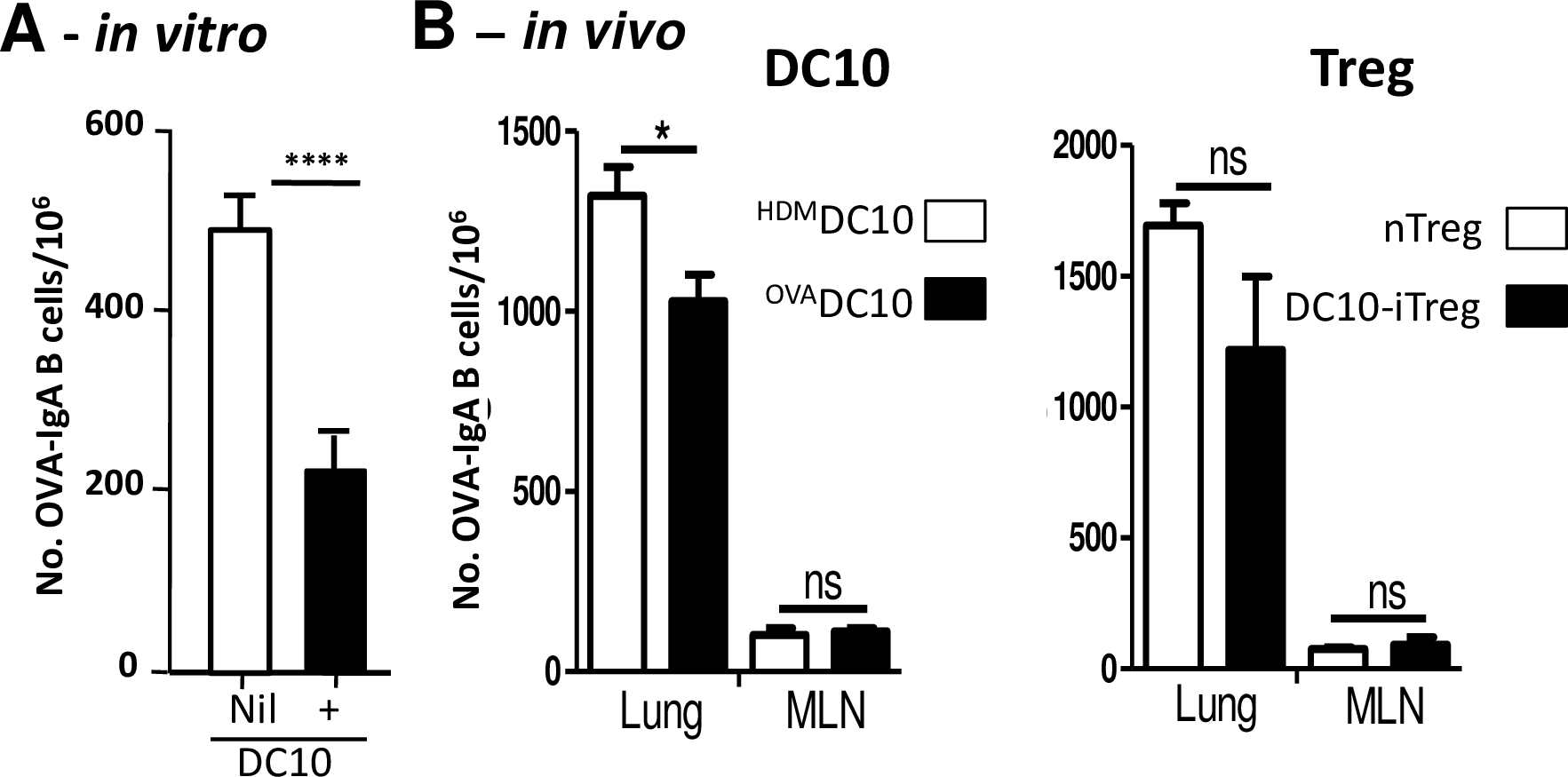

Supplement: S3 Fig — (A) Asthmatic lung single cell suspensions were co-cultured either alone (Nil) or with OVA-pulsed DC10 (lung cell:DC10 ratio of 1:1) for 24 h, and then transferred into ELISPOT plate for 5 h to analyze OVA-specific IgA B cells, as in Fig 3. (B) OVA-loaded DC10 was also injected transtracheally into the airways of asthmatic mice, and 2 dy later single cell suspensions were generated from their lungs and lung-draining (mediastinal) lymph nodes (MLN). Aliquots of the cell suspensions were assayed for OVA-specific IgA-secreting B cells in 5 h ELISPOT assays, as in panel A. DC10 suppressed IgA secretion in vitro, as well as within the lung tissues, but not the MLN of asthmatic mice. We also assessed the impact of natural Treg and DC10-induced Treg on lung and MLN IgA secretion, as was done for IgG1 in Fig 7. Neither the nTreg or iTreg had any impact on the lung or MLN IgA secretion in this assay, although we have reported previously that passive transfer of DC10-iTreg can fully suppress the asthmatic OVA-specific IgE and IgG1 response over 4 wk. The data is presented as the mean number of antibody-secreting cells/106 input cells (±SEM) in 4 wells/sample, with each experiment being repeated 3 times (n = 4 mice/experiment). Statistical analyses were performed using one-way ANOVA assays with Tukey’s post-hoc testing. ** and NS signify p<0.05 and >0.05, respectively. (TIF) [file pone.0190414.s003.tif]
